# Harry Potter and the Osteopathic Medical School: Creating a Harry Potter-Themed Day as a High-Yield Review for Final Exams

**DOI:** 10.1007/s40670-021-01204-2

**Published:** 2021-01-13

**Authors:** Victoria Bryant

**Affiliations:** School of Osteopathic Medicine in Arizona, A.T. Still University, 5850 E. Still Circle, Mesa, AZ 85206 USA

**Keywords:** Harry Potter, Osteopathic medical education, Exam preparation and reviews, Active learning, Wellness activities, Gamification

## Abstract

Incorporating contemporary fiction into educational activities that are interactive and memorable creates a positive learning environment for students. The current article describes how our medical school created a Harry Potter-themed educational event to review didactic material before a final exam. Students were sorted into Hogwarts houses and collected house points in the 8 themed classrooms that reviewed material for the individual disciplines. The event also included a Quidditch tournament and a Yule Ball. The event received positive feedback from students, encouraging the school’s faculty to look for other opportunities to create similar educational experiences during preclinical medical education.

## Introduction

The first year of medical school is a magical time for students, where they gain clinical skills that allow them to treat sick patients with the aim of improving patients’ lives. Similar to medical education, the Harry Potter book series by J.K. Rowling is about a young boy (Harry Potter) who is trying to refine his magical skills and make the world a better place. In *Harry Potter and the Goblet of Fire*, Albus Dumbledore, the Headmaster of Hogwarts School of Witchcraft and Wizardry where Harry Potter learns magic, described how the school could create a community of learners: “We are only as strong as we are united, as weak as we are divided .... Differences of habit and language are nothing at all if our aims are identical and our hearts are open” [1]. At our osteopathic medical school, the students have diverse backgrounds but identical aims of becoming compassionate future physicians, regardless of personal journeys or challenges of matriculation at medical school. One of the tenets of osteopathic medicine is that the person is a unit of body, mind, and spirit [2]; so our school has a strong emphasis on the importance of wellness to achieve this tenet. As such, fun and interactive wellness activities and educational opportunities are integrated into the curriculum to improve the mind, body, and spirit of students and to create a positive learning environment.

In the medical and scientific education fields, contemporary literature, such as the Harry Potter book series, has been used as a teaching tool. In one study, important qualities for clinical teachers to possess were extracted by analyzing the teaching characteristics of the Hogwarts teachers in *Harry Potter and the Philosopher’s (or Sorcerer’s) Stone* [3]. These characteristics are the following: depth of knowledge of their discipline, proficiency in small group facilitation, proficiency in providing feedback, and evaluation of student reasoning [3]. Hogwarts teachers also model professionalism, exhibit creativity in their teaching style, and have flexibility and enthusiasm, all of which can be applied to becoming a successful clinical teacher in the medical school setting [3]. The Harry Potter book series can also be used to enhance the learning experience of students. For instance, difficult genetic concepts like Mendelian inheritance can be introduced to young children using analogies to Harry Potter, linking the unfamiliar concepts with something well known and enjoyable to increase learning and retention [4, 5]. Some have even taken genetic concepts and applied them to the world of Harry Potter to explain the genetic basis of magical traits and the existence of squibs (children who lack magic despite magical parentage) [6]. Similarly, clinicians have applied their medical knowledge to classify the headaches Harry experiences in the books as nummular headaches [7]. Other educators have used the world of Harry Potter to highlight that, just like magic cannot fix every problem, medicine cannot cure every patient [8]. The use of contemporary literature as an educational tool is not limited to Harry Potter; others have used Star Wars to teach about psychopathology [9, 10] or the Hunger Games to teach ultrasound to first-year medical students [11].

Parallels can be drawn between the world of Harry Potter and the world of undergraduate medical education. To be accepted at Hogwarts, students must show some aptitude toward magical abilities. During the medical school admissions process, students must show clinical aptitude as demonstrated by clinical experiences, such as paid employment or volunteering. Over the course of their education at Hogwarts, accepted students learn to become fully qualified practitioners of witchcraft or wizardry but only after successfully passing the required examinations. Likewise, medical students become qualified physicians after completing medical school and successfully passing board examinations. Students at Hogwarts are sorted into 1 of 4 houses by a magical sorting hat, and each house values certain personal characteristics [12]. Mentors during medical school may act as a sorting hat by analyzing a student’s personality, strengths, and weaknesses before suggesting which residency the student should pursue; essentially, the mentor suggests which residency program (house) the student should be sorted into based on shared characteristics with the house [13]. In one study, researchers investigated residency choice as filtered through the Hogwarts house system [14]. While there was some evidence that certain residencies contained a higher number of physicians who self-identified as a certain house, a more comprehensive analysis is needed to determine whether this method is widely applicable or can be used as a predictive tool for residency specialty choice [14].

Gamification involves the application of game design elements in non-game contexts and has been used to engage students in the learning process. It has only recently started to be used in medical education and is a popular addition to curricula [15]. Research suggests gamification increases motivation, engagement, and enjoyment in learning tasks, depending on the context of implementation [16].

Given the benefits of incorporating the world of Harry Potter into educational experiences, we created a Harry Potter-themed educational experience in fall 2018 that included some gamified elements. This event was mandatory for first-year medical students and was intended as a high-yield review of material for their neuromuscular/skeletal/senses (NMSK) final exam. For the final exam of the previous block, we had created a Jeopardy-themed review to prepare students for the exam, and it was very popular. In 2019, enrollment at the school increased from 108 to 162 students, so the Harry Potter event was modified and a second Harry Potter Day was hosted in fall 2019. The event was hugely popular with students and with faculty and staff, and it has become a staple of the curriculum. This event even gained national recognition and was featured in a national organization’s press release [17]. Therefore, the current article describes how we created a Harry Potter-themed educational event to review didactic material before a final exam. This description focuses mostly on the second Harry Potter Day. By sharing information about how this event was created and conducted, other institutions should be able create a similar active and educational experience.

## Creating a Harry Potter-Themed Educational Event

### Introducing the Harry Potter Day to Students

Incoming first-year students were introduced to the themed Harry Potter Day by the school’s upperclassmen during admissions interviews. The event was formally announced in early October when the first-year students received a Hogwarts acceptance letter through Canvas (the school’s online learning platform). During the month before the Harry Potter Day, students could self-identify which Hogwarts house—Gryffindor, Hufflepuff, Ravenclaw, or Slytherin—they wanted to be in by placing their name in a sorting hat box. Students who did not indicate a house preference were randomly assigned to a house, so that each house had an equal number of students. In the Harry Potter series, each Hogwarts house has a professor who is the head of that house, helps represent it, and manages the house Quidditch team. We had 4 faculty members volunteer for this role. Students signed up a month in advance for the house Quidditch teams on a first-come basis. An edition of the *Daily Prophet*, the daily newspaper frequently mentioned in the Harry Potter book series, was created to generate enthusiasm about the event and inform students about the activities of the day. The *Daily Prophet* was distributed to students electronically, and physical copies were available to pick up in the main classroom 2 weeks before the event. In addition, a company was hired to create wristbands in the 4 Hogwarts house colors (red for Gryffindor, yellow for Hufflepuff, blue for Ravenclaw, and green for Slytherin) to remind students of their house. Wristbands also included an inscription about the event. As part of the educational curriculum of the school, approval or exemption by the local institutional review board was not applicable, and students were not required to provide informed consent to participate. However, as much as possible, current ethical standards were followed during the event.

During the week before the Harry Potter Day, some optional wellness-inspired social activities were conducted outside of regularly scheduled class time to allow students to interact with classmates who were also Harry Potter fans. There was an arts and crafts afternoon where students made wands, house banners, and house badges; and they decorated the room that would become the Hogwarts Great Hall for the event. Students invented creative ways of getting into the Hogwarts spirit. In the Harry Potter books, Ravenclaws are known for their intelligence and pursuit of knowledge, so the school’s Ravenclaw students created a “Tree of Knowledge,” which was a blue Christmas tree that students could attach a note to describing a memorable piece of knowledge they had acquired in their medical school journey thus far. There was also a Harry Potter-themed board game night with pizza. In the books, Professor Sprout is the head of Hufflepuff house and teaches Herbology, and our faculty member who was the head of Hufflepuff house provided a herbology lesson at her herb garden. Students picked and dried fresh herbs while learning about their medicinal properties. Attendance at these social events ranged from 12 to 20 students.

### Organization of the Harry Potter Day

In total, 160 of 162 first-year students participated in the 2019 Harry Potter Day; 2 students had excused absences on the day. The participating students were divided equally into 40 students per house, and 112 (70%) preselected their house based on personal preference or by taking an online quiz (there are many personality type quizzes online for sorting people into specific houses) to guide their choice. On the day of the event, each student was given the wristband corresponding to their house as a reminder and as a souvenir. Each student was also given a score sheet so that individual students could record the house points personally collected that day. Students were divided into morning or afternoon sessions because only 80 students at a time could be accommodated in the high-yield exam review. This division was based on preexisting groupings. Furthermore, the review included 8 Hogwarts-themed classrooms, which could accommodate 10 students at a time, so students were randomly assigned to a magical creature group consisting of 10 students per group. The groups were named after the following magical creatures featured in the Harry Potter series: Acromantulas, Basilisks, Blast-Ended Skrewts, Blibbering Humdingers, Bowtruckles, Crumple-Horned Snorkacks, Dementors, Flobberworms, Gulping Plimpies, Hippogriffs, Hungarian Horntails, House Elves, Nifflers, Pygmy Puffs, Thestrals, and Veelas. Students stayed with their group as they rotated through the 8 classrooms. Some classrooms gave out house points to students individually based on how they performed on the activity. Some classrooms had students work as a team, and each member of the magical creature group was awarded the same points. The groups contained a random mixture of houses as this led to the necessary score differences between houses that is needed to award the House Cup to the house with the most points. The morning session was scheduled from 9 am to 12 pm and the afternoon session from 2 pm to 5 pm. The Quidditch tournament was scheduled from 12 pm to 2 pm. For those periods when students were not scheduled to participate in the event, they had free time to study.

### Costumes and the Sorting Ceremony

As part of the Harry Potter Day, faculty, staff, and students were encouraged to dress up. A Google document was created so interested participants could sign up in advance for the character they wanted to dress up as. Faculty and staff dressed up as the following characters: Hogwarts Headmaster Professor Dumbledore, the Minister of Magic, Professor McGonagall, Bellatrix Lestrange, Professor Sprout, Lucius Malfoy, Harry Potter, Professor Snape, Mad-Eye Moody, Fleur Delacour, Madam Pomfrey, Hagrid, and Moaning Myrtle. Students dressed up as the following characters: Draco Malfoy, Petunia and Vernon Dursley, Parvati and Padma Patel, Fred and George Weasley, a mandrake, Dobby, Tonks, Lord Voldemort, Professor Umbridge, Hedwig, Luna Lovegood, and Cho Chang.

The Harry Potter Day allowed students to participate in a Sorting Ceremony, where they were sorted into their Hogwarts house as in the book series. Interested students had to sign up in advance on a Google document to participate. During the ceremony, Dumbledore placed a sorting hat on the heads of the students. Audio clips were played to represent the voice of the Sorting Hat. Once the student’s house was announced, the student was given the wristband for their house and the score sheet for recording house points. Twenty-five (16%) students participated in the Sorting Ceremony.

### Hogwarts-Themed Classrooms

Each subject that was part of the NMSK final exam was assigned to a classroom with a Hogwarts-themed subject. Our school has many small-group study rooms, which are perfect for 10 students to rotate through and contain a large table, a large whiteboard, 2 extra-large computer display screens, and adequate space for seating. Faculty used their own laptops to connect to the computer screens as needed. Some of the faculty decorated their room especially for this event, using handmade decorations or ornaments they owned. We also have a large classroom that can fit approximately 100 people; this room was used as the Hogwarts Great Hall during the event, and it is where we gave instructions and conducted the various ceremonies. The room contains 16 round tables that can fit about 6 people and has multiple whiteboards and computer screens. The students, in their magical creature groups, rotated through the 8 classrooms, spending 20 min per room. A Marauders Map (a magical map that shows the user how to get around Hogwarts) was created to help students navigate to the correct room. A subject-specialist faculty member was assigned to each classroom and created the content for that room based on the pertinent information they wanted to review for the NMSK exam.

The anatomy review classroom (Muggle Studies) used a team competition format, where students earned house points by answering anatomy and embryology questions as they progressed through a Harry Potter-themed fantasy tabletop role-playing game created by the anatomy faculty. This game was highly imaginative and complicated and is briefly described here. A Quidditch LEGO set was used to start, and students teamed up, choosing a Harry Potter LEGO character as a playing piece. Students played a Quidditch game with their playing piece, and certain game actions meant they were asked upper limb anatomy questions. Next, a Whomping Willow LEGO set was used, and players were attacked by a giant spider and had to answer questions about neurological symptoms caused by the spider’s venom. For the final part of the game, students progressed through a checkered dungeon board and were questioned about embryology of limbs and the nervous system as a baby Voldemort developed in a cauldron.

The microbiology review classroom (Fantastic Bugs and Where to Find Them) used a format similar to a Centers for Disease Control and Prevention (CDC) case report, where pairs of students chose 2 cases at random and earned house points by answering questions about the most likely pathogen responsible for those cases. Each case was put into a folder with “MUGGLE CDC – YOUR EYES ONLY” written on it. The case was a clinical scenario describing a patient, their signs and symptoms, and any pertinent medical history or laboratory results. The students then had to identify 3 features of the causative pathogen, name the microbe, and describe how the patient got the infection.

In the physiology review classroom (Physiology of Magic), students raced through a physiology quiz and were awarded house points based on speed and accuracy. In the biochemistry review classroom (Potions), students were individually questioned about biochemical concepts that underlie control of the human mind using open-ended questions. The pharmacology review classroom (Defense Against the Dark Arts) used a competitive format, where a property of a drug (for example, mechanism of action, major side effects) was provided and students had to name the specific medication associated with that property. The opposite order was also used; students were given a specific drug and had to name the queried property. In the clinical review classroom (Care of Magical Creatures), students were asked clinical practice questions of varying difficulty and were individually awarded house points. The questions were created by all the clinical faculty who had taught in the NMSK block. In the lifestyle medicine review classroom (Herbology), students were separated into mini groups and earned house points by answering questions about bone health physiology, pathophysiology, and treatment. The pathology review classroom (The Dark Arts) included a review of questions and high-yield material for the final exam, and the students worked with others in their house to answer questions and earn points.

House points earned in the classrooms were recorded on the students’ score sheets and on whiteboards in each classroom. Points were tallied frequently during the day, so participants could see a running total of the points collected by each house.

### Muggle Quidditch Tournament

As part of the Harry Potter Day, students could also participate in a Muggle Quidditch tournament. In the Harry Potter book series, Quidditch is a sport played on flying broomsticks [12]. Muggle Quidditch has been adapted by our school and by others for play on the ground. There are 7 players on a Quidditch team and 4 player positions (1 Keeper, 1 Seeker, 2 Beaters, and 3 Chasers). The Chasers throw a medium-sized ball called a Quaffle between them and try to score goals through 1 of 3 hoops belonging to the opposing team. Each goal is worth 10 points. The Beaters throw dodgeball-sized balls called Bludgers at the opposing team. If they hit a player on the opposing team with a Bludger, that player is temporarily out of the game (like in dodgeball). The Seeker is the only player on the team allowed to capture the Golden Snitch, which is a small tennis ball-sized ball [12]. Catching the Golden Snitch in Muggle Quidditch is worth 30 points. In the books, the house Quidditch team with the most points is awarded the Quidditch Cup. Instead of playing Quidditch on broomsticks, colored pool noodles were used during our event because they are cheaper and safer and allowed for easy recognition of the different houses. A budget was required to purchase and build Quidditch equipment and was mostly provided by personal donations from faculty members. Suitable balls can be found in any sporting store. We made the hoops out of polyvinyl chloride (PVC) pipes, hula hoops, and medium-density fiberboard (MDF) round wooden bases.

Students who wanted to play on their house Quidditch team signed up on a Google document a month before the tournament. Two outdoor training sessions were provided on campus, so the students could become familiar with the rules. In addition to the 7 required players, each team could have 2 alternates. In total, 36 (23%) students participated, and the teams were required to wear shirts that matched their house color and were encouraged to personalize and decorate the shirts. Because of time constraints, the Quidditch tournament included only 3 Quidditch matches and used a single-elimination format. Each match was 20 min. The Golden Snitch in Muggle Quidditch is a person wearing the tennis ball-sized ball on a waistband, and 3 first-year students who were not on a Quidditch team (1 for each match) volunteered to act as the Golden Snitch.

The Quidditch tournament was scheduled during the 2-h gap between the morning and afternoon sessions and included a pizza lunch for all first-year students. The order of play between house teams was decided randomly by pulling the house names from the Sorting Hat at the beginning of the day, so Hufflepuff played Ravenclaw and Gryffindor played Slytherin in the first 2 matches. The winning teams of those matches (Hufflepuff and Slytherin) played each other in the final. The first 10 min of each match had no Seekers or Golden Snitch. Next, there was a 5-min water break and then the second 10 min was played with the Seekers and the Golden Snitch present. The match ended when a Seeker caught the Golden Snitch or at the end of the 10 min. The team with the most points won regardless of which team caught the Golden Snitch. Several faculty members acted as referees and score keepers. One of the students played the role of Lee Jordan, who was the Quidditch commentator in the book series. The winner of the 2019 Quidditch Cup was Slytherin.

### The Yule Ball

The Harry Potter Day ended with a Yule Ball, where students, faculty, and staff and their families had a potluck dinner. There was even a Hagrid, a half-giant character from the book series, serving Butterbeer. The house points were tallied and finalized at this time, and the winning house was announced and awarded the House Cup by the school’s dean, who was dressed as the Minister of Magic. The 2019 House Cup winner was Gryffindor.

The Yule Ball also included a trivia contest that was intended as a break from the NMSK review questions. Each house had a team and worked together to answer Harry Potter-specific questions. Ravenclaw won this contest.

### Comparison of the First and Second Harry Potter Days

The first Harry Potter Day at our school occurred in fall 2018. It was a full-day event from 8 am to 5 pm with a 2-h break for the Quidditch tournament. From informal feedback, we determined that the students felt this event was too tiring and they would have preferred a half-day event. Because of our increased enrollment in 2019 and student feedback from the first event, for the second Harry Potter Day, we divided the class in half and used 2 sessions, making the event a half-day event from the students’ perspective. During the first Harry Potter Day, we had 12 Hogwarts-themed classrooms, which allowed all 108 students to rotate through in magical creature groups of 9 students per group. During the second Harry Potter Day, since students were divided into 2 groups of 80 students, we reduced the number of classrooms to 8 and had magical creature groups of 10 students per group. This reduction also meant we required fewer faculty volunteers. Furthermore, we added the extra wellness-inspired events, such as the board game night and the herbology lesson. For the first Harry Potter Day, we awarded prizes to individual students with the highest number of house points. However, this delayed awarding of the House Cup because the necessary time to tally points took longer. For the second event, we focused on collective points for the houses only and were able to tally points quicker. As a result and unlike the first event, we were able to award the House Cup on the same day. House points were recorded on whiteboards in each classroom before one magical creature group left and the next entered. Students still kept track of their individual scores on their score sheets but were not required to hand them in. In the future, we would ideally use a technological approach to tally house points, but it would likely still require faculty members to enter points manually to ensure accuracy and fairness.

## Results and Discussion

### Feedback and Future Plans

Immediately after the event, students were asked to complete a voluntary, anonymous electronic survey about the experience. Institutional review board exemption was granted due to the anonymous nature of the survey. The 2 questions on the survey were a Likert scale item about the Harry Potter theme and an open-ended question for written responses. The Likert question asked students whether they enjoyed the Harry Potter theme, using a scale of 1 (strongly disagree) to 5 (strongly agree). Seventy-one students (44% response rate) completed the survey. Of those who completed the survey, 94% agreed or strongly agreed that they enjoyed the Harry Potter theme of the event: specifically, 79% strongly agreed, 16% agreed, 4% neutral, and 1% disagreed.

The open-ended question for written responses received 22 anonymous comments, accounting for 31% of the students who completed the survey. These comments were sorted into the themes of general positive comments (12 comments), comments with suggestions for the future (6 comments), and comments with mixed positive comments and other feedback (4 comments). Examples of general positive comments were the following: “It was very much appreciated and enjoyed and just very nice thank you so much!” and “It was great and was a ton of fun. I personally felt the pressure to start studying (which is what was intended)!” and “HARRY POTTER DAY WAS AWESOME!!!!! I really enjoyed having stations so I could focus on each subject individually and be able to ask that faculty member questions directly. Thank you for such an awesome review!” The 6 comments with suggestions for the future included making the Harry Potter Day a full-day event, having Harry Potter Day sessions throughout the week to increase the time of the review, allowing more than 20 min for each classroom, having more standardized scoring between classrooms, and having students work together to answer questions (this student did not like being put on the spot to answer questions). Since these comments were from individual students, it is unknown whether they represent the majority or minority of students. Logistically, we are limited in the amount of time we can dedicate to this event, so we are unlikely to expand activities in future iterations. The remaining comments with mixed positive comments and other feedback indicated that the students enjoyed the event but it increased their stress levels (2 comments), and there was a lengthy comment that detailed which classrooms the student liked and which ones needed improvement and why.

Some of the students took to social media to discuss the event, using the hashtags #medschoolmotivation, #findingthelight, #feedingthelight, and #enjoystudyingforfinals. One student posted a very positive response on a popular social media platform:

My medical school has a tradition of Harry Potter Day as a high yield final exam review for the neuromuscular block for the entire semester. It was amazing how the teaching faculty, Deans, and docs from the community came together to come in character for Harry Potter Day. We had a sorting ceremony and stations of Harry Potter themes reviews where we reviewed practice questions with professors. There was a lot of laughter and happiness, while maintaining a high-quality review. There was so much sweet thought put into this.

Student interest in Harry Potter-themed medical content continued beyond the Harry Potter Day. For instance, the surgical student interest club conducted a journal club on a recently published article titled, “The Sorting Hat of Medicine: Why Hufflepuffs Wear Stethoscopes and Slytherins Carry Scalpels” [14]. Review of the article prompted a lively discussion on how certain personality traits seem more beneficial in certain medical specialties than others.

The success of our Harry Potter Day is likely due to the immense popularity of the book series and how the characters were brought to life in the movies. Those who discovered the books as children, or even later in life, were given a break from the stressful and serious adult world of medical education. This event also afforded participants the opportunity to immerse themselves in a new world they had only dreamt about before.

Gamification was used in some of our Hogwarts classrooms, notably, the anatomy (Muggle studies) room. Since gamification is becoming increasingly used in medical education and is popular with the current generation of students, future iterations of Harry Potter Day will incorporate more of these elements [15]. After the success of this event, many requests were received from first-year students for other gamified learning events, which are currently under consideration.

### Can This Event Be Conducted Virtually?

Currently, we are in the middle of the COVID-19 pandemic, and our medical school’s curriculum has transitioned to a mostly online format. Therefore, we must consider the future of this annual event and whether it could be transitioned to an online format. Such a change would likely require some high-level Zoom wizardry, such as using Zoom breakout rooms for the classrooms. We are currently exploring a variety of ways to continue this activity during this time.

## Conclusion

This fun and interactive learning experience has become a staple of the curriculum at our medical school. Based on our experiences, several things are necessary for a successful Harry Potter Day. First, a passionate coordinator is necessary to oversee the event. This person must have the Dumbledore-like magical power of making everything work out as planned regardless of the obstacles that inevitably occur. Second, the cooperation of the deans and course directors is necessary, so they support the educational worth of the event and provide time from the curriculum. Third, a sufficient number of faculty have to commit to participate in the event. Fourth, adequate space is mandatory. The Quidditch tournament occurred outside, but it could be moved indoors if there was adequate space. Finally, a budget is necessary for prizes and trophies, Quidditch equipment, decorations, and arts and crafts supplies. Because of limited available school funds, a large part of the budget for our event came from personal donations from the faculty and deans.

This event was a positive and memorable educational experience for our medical students and successfully combined learning with wellness activities. Using the descriptions provided in the current article, other medical schools and undergraduate universities can host similar events like this Harry Potter Day. Before ending this article, in the spirit of the Harry Potter book series and in the words of Dumbledore, “I would like to say a few words. And here they are: Nitwit! Blubber! Oddment! Tweak!” [12].

## Data Availability

Not applicable.
